# The Pop-Gen Pipeline Platform: A Software Platform for Population Genomic Analyses

**DOI:** 10.1093/molbev/msab113

**Published:** 2021-05-05

**Authors:** Andrew Webb, Jared Knoblauch, Nitesh Sabankar, Apeksha Sukesh Kallur, Jody Hey, Arun Sethuraman

**Affiliations:** 1 Center for Computational Genetics and Genomics, Temple University, Philadelphia, PA, USA; 2 Department of Biological Sciences, California State University San Marcos, San Marcos, CA, USA

**Keywords:** population genomics, bioinformatics, primate evolution

## Abstract

The Pop-Gen Pipeline Platform (PPP) is a software platform for population genomic analyses. The PPP was designed as a collection of scripts that facilitate common population genomic workflows in a consistent and standardized Python environment. Functions were developed to encompass entire workflows, including input preparation, file format conversion, various population genomic analyses, and output generation. The platform has also been developed with reproducibility and extensibility of analyses in mind. The PPP is an open-source package that is available for download and use at https://ppp.readthedocs.io/en/latest/PPP_pages/install.html.

Since the advent of genomics, population genetics has become dominated by complex statistical and computational methodologies (Charlesworth and Charlesworth 2016; Casillas and Barbadilla 2017). An unfortunate consequence of this fact is that many investigators lack the necessary resources or time to independently implement many of these methodologies. Investigators must often select from a variety of applications for making particular transformations to data or for conducting particular analyses; and frequently they face unfamiliar input and output formats that may not be intuitive or accessible. These challenges are amplified when analyses require a multi-step pipeline that incorporates a series of transformations and analyses in a specific order.

For investigators working with multiple aligned genomes, these challenges have been partially met by the development of software packages or “tool-kits” that provide for a variety of basic operations and analyses, including vcftools (Danecek et al. 2011), bcftools (Li et al. 2009), and PLINK (Chang et al. 2015). However the functionality of these packages does not extend to more complex transformations and analyses, and their placement in pipelines does not overcome the frequent challenges around reproducibility that arise when implementing complex protocols for population genomic analysis (Mesirov 2010; Baker 2016; Lithgow et al. 2017).

The Pop-Gen Pipeline Platform (PPP) was designed to be an easy-to-use bridge between lower level utilities for manipulating data files and higher level genomic applications. This is achieved through a set of basic Python classes and wrapper scripts that operate on standard data file types, as well as scripts that carry out basic population genomic analyses, and scripts that generate files in formats required by widely used population genomic applications. To demonstrate both the simplicity and the comprehensive nature of the PPP, we designed and implemented a population genomic analysis of publicly available data from chimpanzees (Prado-Martinez et al. 2013) using only the PPP.

## New Approach

### Design

**Fig. 1.**
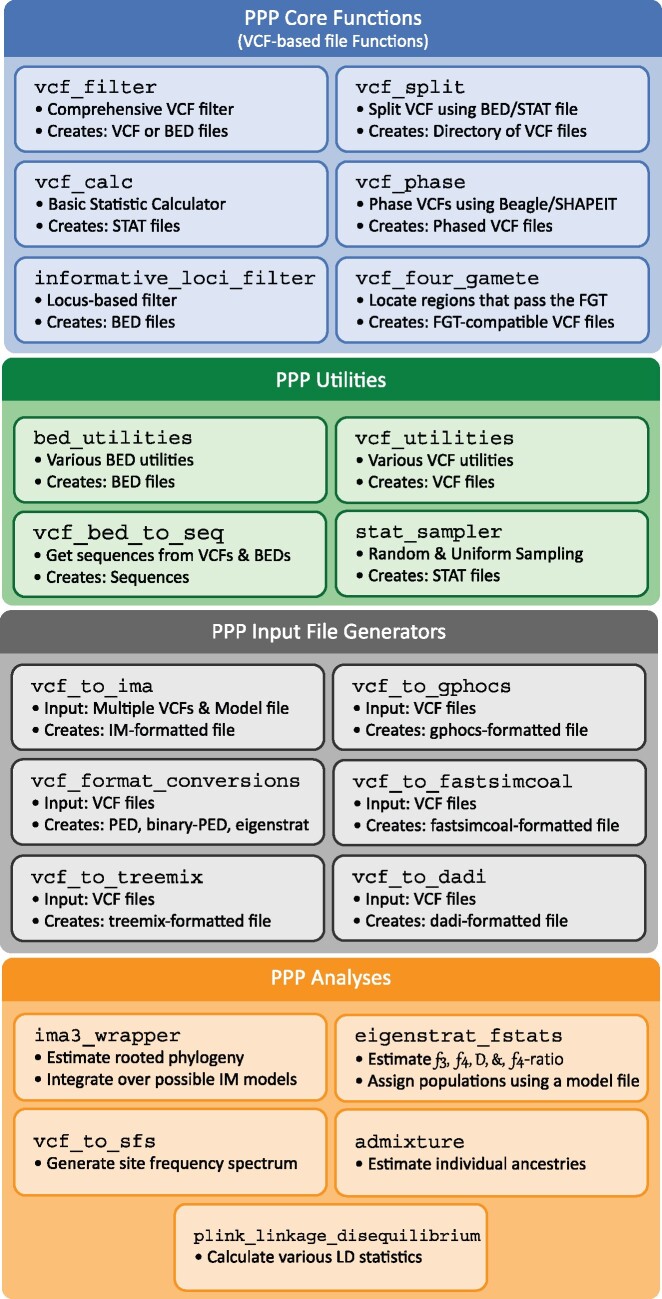
Structure of the PPP. PPP functions are grouped into four categories: 1) the Core PPP functions that operate on VCF files; 2) the optional BED and STAT functions which may be used to sample, filter, and/or edit BED or STAT files; 3) the conversion functions which are required to convert from VCF to analysis-specific file formats; and 4) the analysis functions which are used to automate their respective analyses.

The PPP was written in the Python programming language and designed to operate using Python 3. The PPP was designed as a collection of modular functions (fig. 1) that may be combined to offer a wide variety of analyses and pipelines required by population geneticists. The core functions of the PPP—that is, functions commonly used among analyses—were designed to operate using VCF-based file formats (Danecek et al. 2011). Most runs in the PPP will begin with these core functions, and then branch off into the desired combination of analysis-specific functions. In this way the PPP is intended to help investigators move from general population genomic file formats by providing easy ways to filter and manipulate data and to generate application specific file formats. This design was chosen to avoid superfluous conversions, many of which are computationally intensive. Also, where possible, the PPP integrates frequently used tools, software packages, and statistics, such as the inclusion of both BEAGLE (Browning and Browning 2007) and SHAPEIT (O’Connell et al. 2014) in our phasing function.

To simplify pipeline development for prospective users, we have designed each function to operate within Jupyter notebooks (Kluyver et al. 2016). Jupyter notebooks provide an intuitive and well-documented programming environment that allows code to be written and executed alongside relevant visualizations and text. This enables prospective users of the PPP to easily share entire pipelines, as a notebook may also contain all necessary information for correctly operating the pipeline in addition to displaying plots and figures to examine the output of specific analyses. Another critical feature of Jupyter notebooks, is the ability to separate code—for example, a single function or procedure—into a cell, which allows the separated code to be independently executed. This allows for prospective users to easily examine intermediate results and test code without harming downstream procedures.

A key feature of the PPP is the Model class, which specifies the populations and individuals, assigned to populations, to be used in an analysis. Model information is stored with a JSON-based Model file format, and a Model file can be used to store multiple population models, including the relevant details of each model (i.e., populations, individuals, and other relevant meta-data). A primary benefit of the Model file is the ability to automatically assign information from the specified model to functions, such as the populations and their associated individuals. This file also simplifies record keeping as it becomes the repository for model-related information.

### Example

As shown in table 1, the PPP exists as a set of scripts grouped under “Core Functions,” “Utilities,” “Analyses,” and “Input File Generators” (see also fig. 1). The Utilities scripts provide low level operations on VCF files (sometimes in tandem with a BED file that specifies one or more genomic regions). The CORE Functions scripts provide higher level operations on VCF files, including the generation of data summaries, and four-gamete tests (Hudson and Kaplan 1985). The Analyses scripts will carry out advanced analyses, including linkage disequilibrium calculations, the generation of multi-dimensional site frequency spectra (e.g., as used by ARLEQUIN [Excoffier and Lischer 2010] and fastsimcoal2 [Excoffier and Foll 2011]), conducting Isolation-with-Migration (IM) analyses using IMa3 (Hey et al. 2018), computing f-statistics using EIGENSTRAT (Price et al. 2006), and estimating population structure using ADMIXTURE (Alexander et al. 2009). The Input File Generator scripts can carry out format conversions (e.g., VCF to PED), as well as prepare input files for IMa3 (Hey et al. 2018), G-Pho-CS (Gronau et al. 2011), dadi (Gutenkunst et al. 2009), EIGENSTRAT (Price et al. 2006), ADMIXTURE (Alexander et al. 2009), fastsimcoal2 (Excoffier and Foll 2011), and treemix (Pickrell and Pritchard 2012). Finally, the PPP includes a Model creation script which allows for easy creation of JSON formatted Model files, to be used in conjunction with all the methods described above.

**Table 1:** Comprehensive List of Functions Developed into PPP, Including Filters, File-converters, Data Analyses, and Other Utilities.

Function Type	Script Name	Capabilities
Core (VCF-based)	vcf_filter	*Include/exclude variants sites by:* allele count (i.e., biallelic, multiallelic, invariant), genomic position, missing data count and percentage, MAF, MAC, presence of indels, SNP IDs, and association with a specific flag (i.e., PASS).
Core (VCF-based)	informative_loci_filter	*Include/exclude loci by:* variant site count, missing data count, and locus length. *Control variant count by:* ignoring indels, ignoring multiallelic variants, and ignoring variants within CpG sites.
Core (VCF-based)	vcf_calc	Compute summary statistics from a variant file, including Tajima’s *D*, and Weir and Cockerham’s $F_{\mathrm{ST}}$.
Core (VCF-based)	vcf_split	Uses a BED file of coordinates, or summary statistics to generate separate variant files for each locus or individual.
Core (VCF-based)	vcf_phase	Allows for phasing of variant files by invoking either BEAGLE, or SHAPEIT.
Core (VCF-based)	vcf_four_gamete	Outputs regions of no recombination upon conducting a four-gamete test between pairs of variants. Given phased input with individual variants over a region of the genome, this function generates an interval within those variants that passes the four-gamete filtering criteria, then returns either that interval or an output file with variants in that interval.
PPP utilities	stat_sampler	Computes summary statistics distributions, and pseudorandomly generates subsampled variants/loci either using a uniform sampling scheme, or randomly sampling within bins of statistics.
PPP utilities	bed_utilities	Automates various utilities for BED-formatted files. This currently includes: 1) sample a BED file; 2) subtract from a BED that overlap with a second BED file; 3) extend a BED upstream, downstream, or both upstream and downstream; 4) sort a single BED; 5) merge features within one or more BED files; 6) create a BED of complementary features.
PPP utilities	vcf_utilities	Implements various utilities for manipulation of VCF files, including obtaining a list of the chromosomes within a VCF-based file, obtaining a list of the samples within a VCF-based file, concatenating multiple VCF-based files, merging multiple VCF-based files, and soring a VCF-based file.
PPP utilities	vcf_bed_to_seq	Obtains sequences given a BED coordinates file, and a VCF file.
PPP input file generators	vcf_to_ima, vcf_to_gphocs, vcf_format_conversions, vcf_to_fastsimcoal, vcf_to_treemix, vcf_to_dadi	Conversion scripts that take a variant call format (VCF) file as input, and convert to formats used by IMa3, G-PhoCS, dadi, TREEMIX, and fastsimcoal2.
PPP analyses	eigenstrat_fstats	Contains functions that automate the calculation of multiple admixture statistics, including: Patterson’s D, F4 statistic, F4-ratio statistic, and F3 statistic.
PPP analyses	admixture	Automates the estimation of individual ancestries using Admixture. The functions allows for input as: 1) Binary-PED files or 2) PED 12-formatted files. The function is also capable of configuring the optional arguments of ADMIXTURE.
PPP analyses	ima3_wrapper	Automates the estimation of evolutionary history using IMa3.
PPP analyses	plink_linkage_disequilibrium	Automates the calculation of multiple LD statistics using PLINK.
PPP analyses	vcf_to_sfs	Automates generating the site frequency spectrum (SFS) for a population model from a VCF file.
Model creation	model_creator	Used to produce Model files by either: 1) manually entering the necessary information or 2) by using files with the relevant information. It is also all possible to create multiple models simultaneously and assign populations to more than a single model.

Here, we provide an example of end-to-end pipeline that demonstrates many of the features of the PPP. The starting point is a VCF file (Danecek et al. 2011) that contains multiple aligned genomes from four subspecies of chimpanzees (Prado-Martinez et al. 2013). The endpoint is an IM analysis which includes estimates of population sizes, migration rates, and divergence time. This type of analyses employs a number of assumptions that require careful filtering of the data (Hey and Nielsen 2004). We selected two closely related populations—Central chimpanzees (*Pan troglodytes troglodytes*) and Western chimpanzees (*Pan t. verus*), as the demographic history of the two subspecies has been extensively studied using IM analyses (Won and Hey 2005; Sethuraman and Hey 2016; Chung and Hey 2017; Hey et al. 2018). In particular, we focused on the original IM analyses which used a set of 48 hand-curated loci (Won and Hey 2005) and compared those results with those found using a new data set generated using PPP. Details of the PPP commands are provided in supplementary methods, Supplementary Material online.

**Fig. 2.**
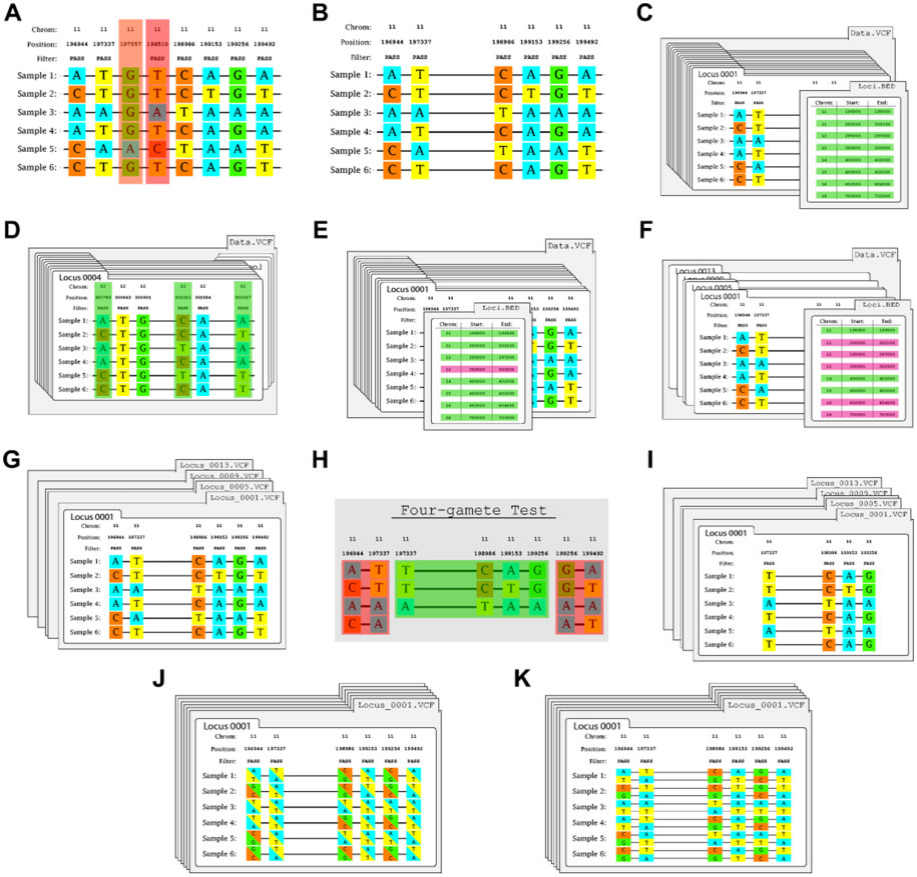
Examples of operations within the PPP. (*A*) Given a VCF, the filtering function identifies two variant sites to remove: 197557 (highlighted in orange) due to not passing all filters—that is, PASS—and 198510 (red) due to being triallelic. (*B*) Once all filters have been applied, a filtered VCF will be produced. (*C*) Many operations require loci (as coordinates) within a VCF to be defined using a BED file. (*D*) Given a VCF and a BED file, the loci-filtering function will confirm that each locus contains at least four variant sites. (*E*) Once all loci-filters have been applied, a filtered BED file will be produced. (*F*) Given a BED file, the bed sample utility may be used to pseudorandomly sample a BED file to reduce the number of loci to specific number. (*G*) The four-gamete test requires each locus to be within a separate VCF, which may be produced using the splitting function. (*H*) Given a locus-VCF, the four-gamete test function is capable of identifying compatible haplotypes. In this example, the haplotypes from 196944 to 197337 and from 199256 to 199492 (highlighted in red) both fail as all possible haplotypes are observed. (*I*) Once the four-gamete test has been applied, compatible locus-VCFs will be produced. (*J*) VCFs may also contain unphased samples, the phasing function using either SHAPEIT or BEAGLE. (*K*) Once the haplotype estimation is complete, a phased VCF will be produced.

The first procedure in our pipeline used the VCF-filter function to select a subset of seven individuals specified in a Model file, and to remove sites unsuitable for our analysis (fig. 2*A* and *B*). VCF-filter is a comprehensive VCF filtering system, with capabilities similar to vcftools (Danecek et al. 2011) and bcftools (Li et al. 2009) (see table 1 for available filters). VCF-filter is especially versatile in being able to simultaneously invoke inclusion and exclusion filters. Using VCF-filter we were able to easily generate a VCF that included only the relevant individuals, excluded the sex chromosomes and sites with missing data, and included only biallelic sites.

We next used the PPP to generate a coordinates file (a BED file) of genomic regions that were at least 10 kb pairs from chimpanzee genes and not-overlapping repetitive and low complexity sequences (see supplementary methods, Supplementary Material online). Following this we used the PPP’s informative-filter to identify regions with sufficient variants for an IM analysis from our coordinates file (fig. 2*C*–*F*). Informative-filter is a loci-filtering function and will remove loci from a BED or PPP-created statistic file if they lack sufficient variants for subsequent analyses. The function may also be configured to define what is counted as variants (e.g., indels, variants within CpG sites) or may be used to remove loci with too much missing data or of insufficient length (see table 1 for details). We then sampled 300 of these regions and generated a VCF file for each one using *vcf_split.py* (see supplementary methods, Supplementary Material online).

Finally, processing of individual loci required haplotype phasing and identification of regions consistent with a lack of recombination following the four-gamete criterion (Hudson and Kaplan 1985). We used VCF-phaser, which invoked the BEAGLE program to generate phased data for each of the sampled loci, as required of IM analyses (fig. 2*J* and *K*). VCF-phaser may be configured to use either SHAPEIT (O’Connell et al. 2014) or BEAGLE (Browning and Browning 2007), and is capable of phasing VCFs, whether they include a single locus or multiple chromosomes. To maintain the versatility of different phasing algorithms, all algorithm-specific options are configurable within VCF-phaser including the ability to specify reference panels.

Like many genealogy samplers, an IM analysis using IMa3 assumes that recombination has happened only between sampled loci (Won and Hey 2005). To help meet this assumption, it is common to sample loci that do not show evidence of recombination (Woerner, Cox, and Hammer 2007; Hey and Wang 2019). We used the PPP’s Four-gamete Test function to generate VCF files of these subsequences (fig. 2*G*–*I*). Four-gamete Test implements the four-gamete test (Hudson and Kaplan 1985) and will identify (where possible) a haplotype block that does not contain variants in intervals that show evidence of genealogy with recombination—that is, a pair of biallelic segregating sites displaying all four possible gametes. The function is highly configurable, including options to require subsequences to include a minimum number of variants, ignore missing data or multiallelic sites, and return either a single or all compatible subsequences. Using Four-gamete Test we were able to limit our locus VCFs to a single subsequence capable with the model assumption of no recombination.

Finally, before proceeding to the IM analysis, we used the PPP’s vcf-to-ima function to convert the individual locus VCFs into a single IM-formatted file compatible with IMa3. vcf-to-ima is a specialized conversion program and is capable of generating an IM-formatted file from collection of VCF files, a Model file, and other configurable model parameters. Once the conversion process was finished, we used the PPP’s ima3-wrapper function to perform an IM analysis. ima3-wrapper is an IMa3 wrapper that is capable of passing all necessary parameters to IMa3, including the number of cores to be used for a parallel run. Using ima3-wrapper we generated an output file with estimates of our desired population model parameters (migration rates, population sizes, and divergence times), with confidence intervals around these estimates.

## Results

To demonstrate the capabilities of the PPP, we generated and analyzed an IMa3 input file with 200 loci from a starting point of a chimpanzee genome VCF file from the Great Ape Genome project (Prado-Martinez et al. 2013). We compared the results for two chimpanzee populations with a previous study that used a set of 48 hand-curated loci that were on average ∼1/4 the length, relative to those generated using PPP (649 base pairs, compared with 2,436) (Won and Hey 2005). We found our estimates of the divergence time, the ancestral chimpanzee population size, migration rates, and the populations sizes of the extant chimpanzee populations—Central chimpanzees (*P. t. troglodytes*) and Western chimpanzees (*P. t. verus*) to be very close to the previous study, including the finding of significant gene flow from *P. t. verus* to *P. t. troglodytes* (table 2).

**Table 2:** Evolutionary History of Central and Western Chimpanzees, Estimated Using PPP and IMa3.

Subspecies	Analysis	Parameter^a^	Estimate^b^	95% Confidence Interval
*Pan troglodytes troglodytes*	IM	*N*	33,640	24,26054,601

	PPP, IMa3	*N*	59,911	51,74371,509

* Pan troglodytes verus*	IM	*N*	9,187	6,33314,196

	PPP, IMa3	*N*	9,270	8,37210,414

* Ancestor*	IM	*N*	6,303	61417,092

	PPP, IMa3	*N*	7,474	5,922-9,107

	IM	*t*	758,504	495,003-1,390,904

	PPP, IMa3	*t*	740,662	668,662-829,212

*P. t. t.* →*P t. v.*	IM	*m*	6.83E–08	1.07E–07
				1.25E–05
	PPP, IMa3	*m*	1.89E–06	3.79E–06
				3.01E–07
	IM	*2Nm*	0.00126	—
	PPP, IMa3	*2Nm*	0.03314	—
*P. t. v.* →*P. t. t.*	IM	*m*	7.79E–06	2.40E–06
				1.93E–05
	PPP, IMa3	*m*	2.63E–06	4.47E–06
				9.56E–07
	IM	*2Nm*	0.5243	—
	PPP, IMa3	2*Nm*	0.2995	—

a Estimates for intervals for population size (*N*), splitting time (*t* in years), migration rate (*m* per gene copy per generation), and population migration rates (*2Nm*) scaled by the geometric mean of mutation rates. IM (Hey and Nielsen 2004) results were obtained from the first IM analysis of *P. t. troglodytes* and *P. t. verus* (Won and Hey 2005), which used 48 hand curated loci and the original IM program (Hey and Nielsen 2004).

b Maximum likelihood estimates. To convert primary parameter estimates, which are scaled by the geometric means of mutation rates, we used the geometric mean of locus-wide mutation rates per year, assuming a per year rate of 1.2*e*-8 per base (Scally and Durbin 2012) and 24.5 years per generation (Langergraber et al. 2012). For the 48 loci in the original IM study, the value was 2.65654E–07 per year, whereas the corresponding rate was 9.97486e–07 for the longer loci sampled using PPP.

## Discussion

The primary goal behind the development of the PPP was to create an accessible platform for population genomic analyses; one that investigators could use to easily get from sets of aligned sequences (as VCF files) to the carrying out of complex downstream applications applied to one or more user-defined sampling models. To demonstrate, we examined the demographic history of two chimpanzee subspecies and compared the results to previous findings (Won and Hey 2005; Chung and Hey 2017; Hey et al. 2018). We found that the PPP greatly simplified the generation of IMa3 input files. Assembling the pipeline was a straightforward process as the majority of functions could be invoked in tandem without requiring intermediate processing steps. We were also able to quickly process the VCF input for our IM analysis as the majority of PPP functions required <5 min to operate, with the exception being the initial filtering procedure which took roughly 50 min. The IMa3 run on 200 loci required ∼40 h on 20 CPUs. We also found that repeating our analysis—either to explore the results of different parameters, reproduce our findings, or remedy errors—was a simple process.

Although our overview of the PPP focused on performing an IM analysis, the PPP was designed to easily allow the implementation of many other analyses. For example, we could easily modify our example IMa3 pipeline to generate input files for studying population structure using ADMIXTURE (Alexander et al. 2009), or test for introgression using AdmixTools (Patterson et al. 2012), or linkage disequilibrium using PLINK (Chang et al. 2015). Additionally, PPP includes functions for running a variety of commonly used population genomics tools, including EIGENSTRAT (Price et al. 2006) and generate site frequency spectrum (SFS) data that can be extended to tools, such as fastsimcoal2 (Excoffier and Foll 2011) and ARLEQUIN (Excoffier and Lischer 2010).

It is also possible to operate analyses external to the PPP by directly calling an executable or creating a script, both of which could ideally be shared using a Jupyter notebook. Examples of Jupyter notebooks to run PPP analyses have been described in the online documentation and GitHub pages for the project. To promote extensibility of the pipeline, we invite the population genetics community to contribute toward developing additional tools into PPP. Details on contributing to the project are described at https://ppp.readthedocs.io/en/latest/index.html.

## Supplementary Material

Supplementary data are available at *Molecular Biology and Evolution* online.

## Supplementary Material

msab113_Supplementary_Data
