# Supplementary material for: The Pop-Gen Pipeline Platform: A Software Platform for Population Genomic Analyses
Source: Mol Biol Evol. 2021 May 5;38(8):3478–85. doi: 10.1093/molbev/msab113 (PMC8321520; doi:10.1093/molbev/msab113)
Supplement: msab113_Supplementary_Data [file msab113_supplementary_data.zip › PPP_MBE_Supplementary.pdf]

Supplementary Methods

Initial Filtering of Chimpanzee Dataset

We began by generating a Model file for 2 populations, including three Central chimpanzees (*Pan troglodytes troglodytes*) and four Western chimpanzees (*Pan troglodytes verus*) that were sequenced to high coverage as part of the the Great Ape Genome Project (Prado-Martinez et al. 2013). We used **vcf_filter.py** to generated a VCF file for only these individuals, and to remove non-biallelic variant sites, variant sites with missing data, and variants on the sex chromosomes using the following command. The removal of non-biallelic sites (i.e. multi-allelic sites) is of particular importance as they violate the Infinite Sites (IS) model (Kimura 1969) assumption of a single polymorphism per site assumed by the IM model Hey and Nielsen (2007) implemented in our analysis pipeline. This operation, done in a single step, reduced the total number of variants from 2,681,597,652, in the original VCF file, to 1,478,942,393 in the filtered file.

```
vcf_filter.py --vcf Pan_troglodytes.vcf --model-file PttPtv.model --model
     2Pop --out 2Pop.TV.Filtered.vcf.gz --filter-only-biallelic --filter
    -max-missing 0.0 --filter-exclude-pos chrX chrY chrM
```

Filter for non-genic regions without repeats

The Great Ape Genome project VCF files are based on human reverence NCBI36 (aka hg18). We downloaded BED files from the UCSC table browser (Kent et al. 2002) for NCBI36 RefSeq genes (including 10000 bases flanking on either side) and for repeats identifed using RepeatMasker (Smit et al. 2013-2015). Region within 10kb of the genes were targeted for removal as the IM pipeline required variant sites to be putatively neutral (Hey and Nielsen 2007). Repeat sequences were avoided because of a higher chance of misalignment in genome assembly. We used **bed_utilities.py** in four steps to generate a BED file of intervals that do not contain either genes or repeats, and that intersect with the VCF file. First we merged the two BED files. Second we generated a BED file with intervals of 3000 base pairs, a length expected to be longer than any sampled loci based on the four-gamete criterion, given previous experience with chimpanzee genomes (Hey et al. 2018; Won and Hey 2005). Third we used **bed_utilities.py** to subtract from the 3 kb interval BED file, those intervals that overlapped with genes or repeats. Finally we identified those regions that include the 3 kb windows not intersecting with genes or repeats, and that intersect with the VCF file.

```
bed_utilities.py --utility merge --beds merge hg18_genes.10kb_flank.bed
    hg18_RM.bed > hg18_genes_RM.bed

bed_utilities.py --utility windows --out hg18_windows.bed --chrom-file
    hg18.chrom.sizes_NoMXY --window-size 3000
```

```
bed_utilities.py --utility subtract --bed hg18_windows.bed --subtract-bed
     hg18_genes_RM.bed --subtract-entire-feature --out hg18_windows.
    genes_subtracted.bed
```

```
bed_utilities.py --utility intersect --bed hg18_windows.genes_subtracted.
    bed --intersect-file 2Pop.TV.Filtered.vcf.gz --out
    hg18_windows_intersects.genes_and_RM_subtracted.bed
```

Identification, Filtering, and Sampling Chimpanzee Loci

We next used **informative_loci_filter.py** to identify regions in the VCF files that had no-missing data at variable sites and that had at least four phylogenetically informative sites. The four variant threshold was used to avoid invariant regions. We then randomly sampled 300 regions using **bed_utilities.py** and made a separate VCF file for each region using **vcf_split.py**.

```
informative_loci_filter.py --vcf 2Pop.TV.Filtered.vcf.gz --bed
    hg18_windows_intersects.genes_and_RM_subtracted.bed --remove-indels
    --minsites 4 --keep-full-line --out hg18_windows_informative.
    genes_and_RM_subtracted.bed
```

```
bed_utilities.py --utility sample --bed hg18_windows_informative.
    genes_and_RM_subtracted.bed --sample-size 300 --out hg18_sampled.
    genes_and_RM_subtracted.bed --random-seed 123
```

```
vcf_split.py --vcf 2Pop.TV.Filtered.vcf.gz --split-method bed --split-
    file hg18_sampled.genes_and_RM_subtracted.bed --out-dir 2
    Pop_TV_sampled --out-prefix 2Pop.TV
```

Phasing, and application of four-gamete filter, and final selection of loci

We next phased the loci VCFs with BEAGLE (Browning and Browning 2007) using the default parameters of the algorithm. Each locus was phased individually using the following command.

```
vcf_phase.py --vcf 2Pop_TV_sampled/2Pop.TV_0.vcf.gz --phase-algorithm
    beagle --random-seed 123 --out 2Pop_TV_phased/2Pop.TV_0.vcf.gz
```

We then used **vcf_four_gamete.py** to identify intervals that pass the four-gamete criterion and to sample one at random for each locus. We used settings on the four-gamete filter (–numinf 3 –reti –right) to select a random non-overlapping interval with a minimum of three phylogenetically informative variable sites. It is important that non-overlapping intervals be selected, when filtering on the basis of the four-gamete criterion, so as not to bias the results (Hey and Wang 2019).

```
vcf_four_gamete.py --vcfs 2Pop_TV_phased/2Pop.TV_0.vcf.gz --fourgcompat
    --reti --right --numinf 3 --out 2Pop_TV_fgt/2Pop.TV_0.vcf
```

IM File Creation and Analysis

We next converted the sub-region VCFs from the four-gamete test into a single IMa3-formatted file using **vcf_to_ima.py**. This operation requires a mutation rate per base pair per year. We based this rate on 1.25e-8 per base per generation (Scally and Durbin 2012) and a generation time of 24.5 years Langergraber et al. (2012), to yield a per year mutation rate of 4.92e-10. The output file had 273 loci that passed the four gamete filter, and this was trimmed down to 200 loci in a text editor.

```
vcf_to_ima.py --vcfs @4gamete_files.txt --model-file PttPtv.model --model
     2Pop --out Ptt_Ptv_alt_4.92em10_200L.u --mutrate 4.92e-10
```

The **ima3_wrapper.py** script was used to run IMa3 on 20 cores for approximately 40 CPU hours using the following command. The priors for population sizes, migration rates and splitting time were -q8, -m 1.0, and -t 1.5, respectively. The heating terms (200 chains, with geometric heating terms -ha0.994 -hb0.50) were as suggested in the IMa3 documentation (Hey et al. 2018). The program was run for a 20 hour burn-in, followed by a 20 hour sampling period which generated 87,425 sampled genealogies per locus.

```
ima3_wrapper.py --threads 20 --ima-path IMa3
 -b 20.0 -L 20.0 -q8 -m 1.0 -t 1.5 -u24.5 -hn200 -ha0.994 -hb0.50 -i
     Ptt_Ptv_alt_4.92em10_200L.u -o Ptt_Ptv_alt_4.92em10_200L.out
```

References

Browning SR, Browning BL. 2007. Rapid and Accurate Haplotype Phasing and Missing-Data Inference for Whole-Genome Association Studies By Use of Localized Haplotype Clustering. Am. J. Hum. Genet. 81:1084–1097.

Hey J, Chung Y, Sethuraman A, Tishkoff S, Lachance J, Sousa VC, Wang Y. 2018. Phylogeny Estimation by Integration over Isolation with Migration Models. Mol. Biol. Evol. 35:2805–2818.

Hey J, Nielsen R. 2007. Integration within the Felsenstein equation for improved Markov chain Monte Carlo methods in population genetics. Proc. Natl. Acad. Sci. USA 104:2785–2790.

Hey J, Wang K. 2019. The effect of undetected recombination on genealogy sampling and inference under an isolation-with-migration model. Mol. Ecol. Resour. 19:1593–1609.

Kent WJ, Sugnet CW, Furey TS, Roskin KM, Pringle TH, Zahler AM, Haussler D. 2002. The human genome browser at ucsc. Genome Res 12:996–1006.

Kimura M. 1969. The number of heterozygous nucleotide sites maintained in a finite population due to steady flux of mutations. Genetics 61:893–903.

Langergraber K, Prüfer K, Rowney C, Boesch C, Crockford C, Fawcett K, Inoue E, Inoue-Muruyama M, Mitani J, Muller M. 2012. Generation times in wild chimpanzees and gorillas suggest earlier divergence times in great ape and human evolution. Proc. Natl. Acad. Sci. USA 109:15716–15721.

Prado-Martinez J, Sudmant PH, Kidd JM, Li H, Kelley JL, Lorente-Galdos B, Veeramah KR, Woerner AE, O'Connor TD, Santpere G, et al. 2013. Great ape genetic diversity and population history. Nature 499:471–475.

Scally A, Durbin R. 2012. Revising the human mutation rate: implications for understanding human evolution. Nat. Rev. Genet. 13:745–753.

Smit AFA, Hubley R, Green P. 2013-2015. Repeatmasker open-4.0.

Won YJ, Hey J. 2005. Divergence population genetics of chimpanzees. Mol. Biol. Evol. 22:297–307.
